# Evaluating the Therapeutic Potential of Common Sage (*Salvia officinalis* L.): Antioxidant Capacity, Cytotoxicity and Dose-Dependent Effects on Gastric Smooth Muscle Viability and Function

**DOI:** 10.3390/molecules31152647

**Published:** 2026-07-29

**Authors:** Ekaterina Zaytseva, Raina Ardasheva, Natalia Prissadova, Viktor Yotov, Iva Slavova, Teodora Tomova, Stanislav Dyankov, Mariana Argirova, Athanas Krastev

**Affiliations:** 1 Department of Medical Physics and Biophysics, Faculty of Pharmacy, Medical University of Plovdiv, 4002 Plovdiv, Bulgaria; ekaterina.zaytseva@mu-plovdiv.bg (E.Z.); rayna.ardasheva@mu-plovdiv.bg (R.A.); nataliya.prisadova@mu-plovdiv.bg (N.P.); athanas_kristev@yahoo.com (A.K.); 2 PERIMED-2, BG16RFPR002-1.014-0007, Central District, Vasil Aprilov Blvd. 15A, 4002 Plovdiv, Bulgaria; iva.slavova@mu-plovdiv.bg (I.S.); teodora.tomova@mu-plovdiv.bg (T.T.); stanislav.dyankov@mu-plovdiv.bg (S.D.); 3 Department of Chemical Sciences, Faculty of Pharmacy, Medical University of Plovdiv, 4002 Plovdiv, Bulgaria; mariyana.argirova@mu-plovdiv.bg; 4 Department of Pharmacognosy and Pharmaceutical Chemistry, Faculty of Pharmacy, Medical University of Plovdiv, 4002 Plovdiv, Bulgaria; 5 Research Institute, Medical University of Plovdiv, 4002 Plovdiv, Bulgaria

**Keywords:** *Salvia officinalis*, antioxidant activity, cytotoxicity, rosmarinic acid, gastric smooth muscle, traditional medicine

## Abstract

*Salvia officinalis* L. (*S. officinalis*) or common sage has long been valued in traditional medicine for its broad therapeutic properties. Our study explored the antioxidant potential, the cytotoxic activity and the effects on smooth muscle (SM) contractility of a traditionally prepared sage aqueous infusion. A chromatographic analysis revealed the major phenolic constituents of the infusion, rosmarinic acid being the most abundant compound (38.16 mg/mL), followed by rutin (3.58 mg/g), caffeic acid (3.00 mg/g) and hesperidin (1.84 mg/g). Three spectrophotometric methods were used to determine the antioxidant potential of the plant extract: a DPPH assay for radical scavenging activity (IC_50_ 21.77 ± 0.98 µg/mL), a CUPRAC assay (2.32 ± 0.04 mmol Trolox equivalents/g) and a total reducing capacity assay (1.92 ± 0.01 mmol Trolox equivalents/g) for evaluating reducing properties. A cytotoxicity was assessed in primary SM cells (isolated from rat gastric tissue) using the xCELLigence real-time cell analysis system, where the infusion demonstrated dose-dependent cytotoxicity with an IC_50_ of 0.34 ± 0.12 mg/mL. Concentrations of 0.37 mg/mL rapidly reduced viability, while lower concentrations showed mild or negligible effects. Functional experiments with isolated rat gastric SM cells revealed a dose-dependent enhancement of both tonic and phasic contractile activity. Our results showed that the serotonergic pathways were mainly implicated in mediating these effects, while the cholinergic pathways were not involved with them. Together, these findings validate the traditional use of *S. officinalis* and point to its role in modulating SM function and providing antioxidant benefits, warranting further investigation into its mechanisms and therapeutic potential.

## 1. Introduction

Medicinal and aromatic plants and their crude preparations have been used since ancient times for their biological properties and various health benefits [1,2,3,4]. *Salvia officinalis* L. (common sage, *S. officinalis*) is one of nearly 1000 known species of the genus Salvia, belonging to one of the largest plant families, Lamiaceae [5,6]. Its traditional medicinal use is well documented and dates back thousands of years. Although the plant is native to the Mediterranean region, due to its diverse health benefits, it is also subject to cultivation in many European countries including Bulgaria [7]. Bulgarian folk medicine uses mainly the plant leaf extracts in different forms: as an infusion, topical ointment or tincture, for inhalation, and even by chewing fresh leaves [8]. These preparations have been used to treat oral infections, inflammation of the respiratory or gastrointestinal tract, as an estrogenic agent to attenuate some postmenstrual vegetative disorders, or as a hypoglycemic remedy [8]. Along with these plant benefits claimed by traditional medicine, nowadays, many scientific studies not only confirm but also expand its potential health benefits profile.

Many members of the polyphenol secondary metabolite group have been found in *S. officinalis* aqueous, organic, or mixed extracts, whose type and concentration may vary depending on the geographical origin and the type of solvent used. The polyphenols present in plants are categorized within two major groups: flavonoids and phenolic acids. They are most often glycosylated, which increases their solubility in water, but the variety of the carbohydrate moiety included in their structure contributes to the large diversity of these plant metabolites. The phenolic compounds present in *S. officinalis* plant extracts, such as rosmarinic acid (RA), exhibit a wide range of biological activities including anti-inflammatory, antimutagenic, cytotoxic, antimetastatic, neuroprotective, antimicrobial, immunomodulatory, and melanogenic activities [9].

In addition, certain plant polyphenols have been reported to modulate smooth muscle (SM) function through several molecular mechanisms, exhibiting either spasmolytic (relaxing) or prokinetic (contracting) effects, depending on the tissue and the signaling pathways involved [10,11,12,13,14]. Experimental studies suggest that these compounds influence membrane ion channel kinetics and receptor-mediated signaling, and they are involved in gastrointestinal regulation, although these effects appear to be indirect and remain incompletely understood [13,15,16]. Among the phenolic constituents of *S. officinalis*, RA has been shown to exhibit acetylcholinesterase inhibitory activity, suggesting an indirect influence on cholinergic neurotransmission rather than a direct interaction with muscarinic receptors [10,16,17]. Furthermore, RA has been reported to affect SM ion channels such as voltage-gated Ca^2+^ channels (blocking influx) and K^+^ channels (promoting membrane hyperpolarization) [10,13,14]. Most studies have been performed in neuronal, vascular, or intestinal models, whereas the effects of *S. officinalis* and its major phenolic constituents on stomach SM contractility and the proliferation of stomach SM cells remain insufficiently characterized. Furthermore, the biological activity of *S. officinalis* extracts reflects the combined actions of numerous phytochemicals that may exert complementary or opposing effects on gastrointestinal motility, depending on their relative composition and concentration.

In recent years, extracts and essential oils, including those of *S. officinalis*, have been the subject of intensive research to document their traditional uses and to identify new biological effects [18,19,20]. We undertook this study to expand the knowledge about the effects of *S. officinalis* on its ability to influence gastric muscle cell proliferation and muscle activity. Gastric SM tone and motility are regulated by multiple receptor types responding to neural (enteric/autonomic), hormonal, and paracrine signals. The key receptors are muscarinic M_3_, adrenergic, serotonin, and dopamine receptors. The experiments were carried out with an infusion prepared according to traditional medicine. This type of plant extract has the advantages that it can be prepared daily at home and its preparation does not require the use of specialized equipment, unlike water–alcoholic tinctures or essential oils.

## 2. Results

### 2.1. Total Phenolic Content and Chromatographic Analysis

The total content of phenolic compounds in the infusion used in this study was 208 ± 10.9 mg gallic acid equivalents (GAEs)/g dry matter, which is higher than the previously reported findings [21].

**Figure 1:**
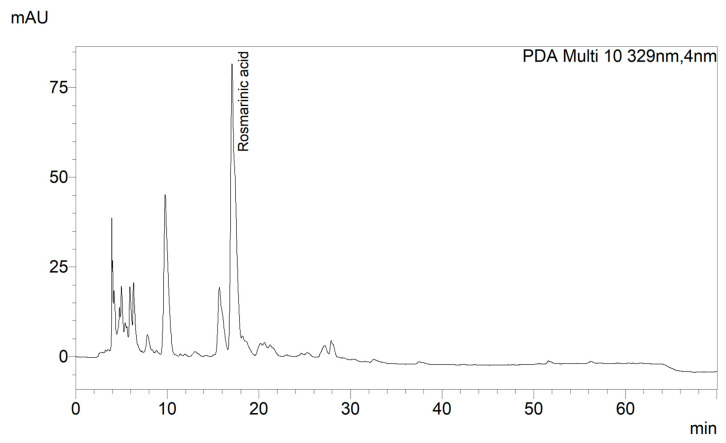
HPLC-PDA chromatogram of *S. officinalis* infusion at the absorption maximum of RA (329 nm).

The phenolic profile of the *S. officinalis* infusion was further assessed by high performance liquid chromatography with a photodiode array detector (HPLC-PDA). The infusion was screened for the presence of fifteen common plant phenolic secondary metabolites, as listed in Table 1. RA was the most abundant phenolic acid present in the infusion. Among the flavonoids, two glycosides were identified: rutin, with a concentration of 3.58 mg/g, followed by hesperidin (1.84 mg/g). A representative chromatogram used for the quantification of RA is presented in Figure 1.

### 2.2. Antioxidant Assays

The high content of phenolic compounds in the infusion may be a prerequisite for the presence of antioxidant properties with potential therapeutic effects. Oxidative stress is recognized as an important contributor to gastrointestinal dysfunction, as excessive reactive oxygen species (ROS) impair SM function, alter intracellular signaling pathways, and interfere with cell proliferation [22,23]. Therefore, the antioxidant activity of the infusion was evaluated using different methodologies. The antioxidant activity of the sage infusion was determined by two methods based on the single electron transfer mechanism (total reducing capacity and cupric ion reducing antioxidant capacity assays) and by the DPPH method, based on the ability of the infusion to transfer both hydrogen atoms (HAT mechanism) and single electrons (SET mechanism) (Table 2).

As shown in Table 2, the *S. officinalis* infusion was effective in scavenging DPPH radicals. In addition, the strong antioxidant activity of the infusion was also confirmed by the results of the CUPRAC and the total reducing capacity (TRC) assays, demonstrating the very good reduction properties of the infusion, with respect to both Cu^2+^ and Fe^3+^ ions.

### 2.3. In Vitro Cytotoxicity Investigation

To evaluate the cytotoxic effects of an aqueous infusion of *S. officinalis* on a primary cell culture derived from rat stomach SM, cells were treated with various concentrations of the infusion: 2.45, 0.98, 0.73, 0.49, 0.37, 0.25, 0.12, 0.061, 0.031, 0.01531, 0.0077 and 0.0038 mg/mL. Cell viability was monitored in real time using the xCELLigence system over a period of 48–72 h.

**Figure 2:**
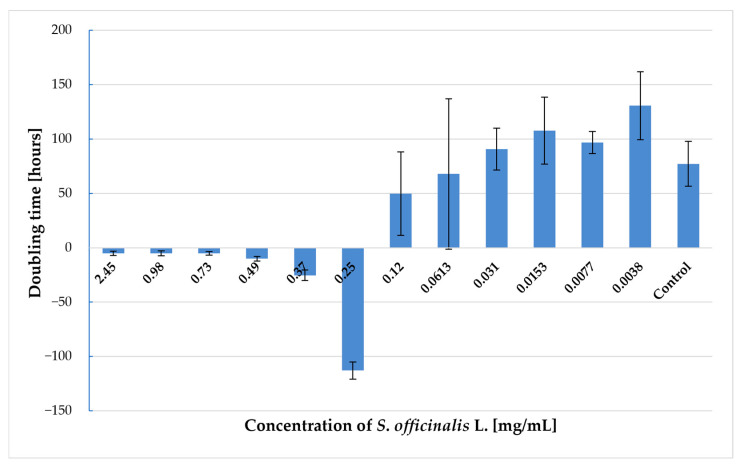
Dependence of the monolayer doubling time on the applied concentration of *S. officinalis*. The values are calculated from xCELLigence RTCA-DP software (2.0.0.1301).

The results obtained on the rat SM cells demonstrated acute cytotoxicity at infusion concentrations of 0.37 mg/mL and higher. Under these conditions, the Cell Index (CI) decreased rapidly, reaching values below zero within 3–5 h, indicating the loss of vital adhered cells in the wells of the culture plate. In addition, the software-calculated doubling time (DT) of the cell population in the treated wells was negative and markedly short (Figure 2).

**Figure 3:**
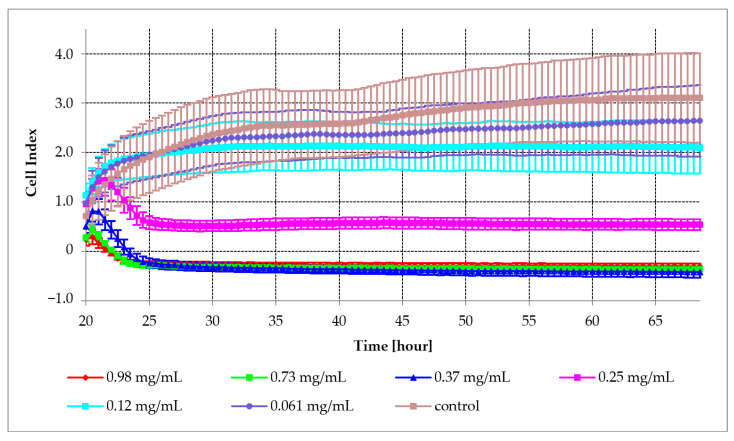
Evolution of Cell Index over time, showing the development of the monolayers after treatment with different concentrations of *S. officinalis* infusion.

Treatment with the *S. officinalis* infusion at a concentration of 0.25 mg/mL resulted in an increased DT value. Although the DT remained negative, it was considerably longer than that observed at higher concentrations, suggesting a reduction in the cytotoxic effect. Figure 3 presents the results of the real-time CI monitoring, which demonstrated a marked decrease in the CI value. Despite this decline, the CI remained positive throughout the experiment, indicating the persistence of viable cells attached to the bottom of the wells. However, cell proliferation was suppressed, and a confluent cell monolayer failed to develop.

Similarly, treatment with the infusion at a concentration of 0.12 mg/mL inhibited cell proliferation without inducing pronounced cytotoxic effects—the CI remained relatively stable but did not reach the values observed in the untreated control cell population. At concentrations below 0.12 mg/mL, the *S. officinalis* infusion had no detectable effect on cell growth dynamics or monolayer formation, as the results were comparable to those of the control group (Figure 2).

Notably, large standard deviations were observed for the wells treated with 0.12 mg/mL and 0.0613 mg/mL infusion. The relatively high variability observed in the DT is likely attributable to the algorithm used by the xCELLigence RTCA software(2.0.0.1301) for the DT calculation. Since the DT is derived from the slope of the CI curve during the exponential growth phase, the calculated values become highly sensitive to minor fluctuations in the CI under conditions of markedly reduced proliferation or a nearly stable CI. This limitation is particularly evident at concentrations near the transition between antiproliferative and cytotoxic effects. Furthermore, primary SM cells constitute a heterogeneous population with variable proliferative capacities, which may further contribute to the observed variability.

### 2.4. Effect of S. officinalis Infusion on Tonic and Spontaneous Contractile Activity of Gastric Smooth Muscles

The *S. officinalis* aqueous infusion causes a dose-dependent contractile tonic effect. When its concentration in the tissue bath is increased by the successive addition of final volumes (final concentrations in the tissue bath of 0.245 mg/mL, 0.49 mg/mL and 1.96 mg/mL), the amplitude of contraction increases. The maximum value for each concentration is presented in Table 3.

Based on the results, the next experiments were performed with *S. officinalis* in a final concentration in the tissue bath of 1.96 mg/mL. A single exposure at this concentration caused a contractile SM response of 4.12 ± 0.19 mN (n = 8).

Exposure to the infusion increased the amplitude of spontaneous phasic contractions, which increased with time. After 30 min of exposure, the strength of the phasic contractions significantly decreased relative to their auto-control values (Table 4).

Treatment with the infusion does not affect the frequency of the spontaneous contractions, as it remains within 4.5–5 contractions/min.

### 2.5. Mutual Influences on the Spontaneous Activity of SM Between an S. officinalis Infusion and Elements of Some Neurotransmitter Chains

#### 2.5.1. Mutual Influences on the Spontaneous Activity of SM Between an Aqueous Infusion of *S. officinalis*, Acetylcholine (ACh) and Blocker of Muscarinic Cholinergic Receptors (m-AChR)

In the presence of 1.96 mg/mL of the *S. officinalis* infusion, the strength of 1 × 10^−6^ mol/L ACh-induced SM contractions did not differ compared to the control (Table 5).

In another set of experiments, in the background of 1 × 10^−6^ mol/L ACh-induced SM contraction, 1.96 mg/mL of the infusion provoked an additional contractile effect, independent of the pathway through which ACh induces a contraction. The quantitative data are shown in Table 6. The results show the presence of statistically significant differences (t = 8.742; *p* < 0.001; df = 6).

In proof of the above, experiments were performed with two blockers of m-AChR in the SM. Blocking the m-ACh receptors with 1 × 10^−6^ mol/L 1,1-dimethyl-4-diphenylacetoxypiperidinium iodide (4-DAMP) or with 1 × 10^−6^ mol/L atropine, selective muscarinic receptor antagonists, did not significantly affect the magnitude of the infusion-induced (1.96 mg/mL) contractile SM responses (Table 7).

#### 2.5.2. Mutual Influences on the Spontaneous Activity of SM Between an Aqueous Infusion of *S. officinalis*, 5-Hydroxytryptamine (5-HT) and 5-HT Receptor Blocker

5-HT-induced contractile responses (1 × 10^−6^ mol/L) in the presence of the *S. officinalis* infusion were significantly reduced (t = 7.014; df = 7; *p* < 0.001). The maximum values of the control and in the background of 1.96 mg/mL of the infusion are shown in Table 8.

After blocking the 5-HT receptors with 3 × 10^−7^ mol/L Methysergide, the *S. officinalis* infusion caused significantly weaker contractions (t = 13.508; df = 3; *p* = 0.001) (Table 9).

## 3. Discussion

Phenolic compounds are often the main contributors to the antioxidant capacity of plant-based extracts. RA, an ester of caffeic acid and 3,4-dihydroxyphenylacetic acid, which is the principal phenolic constituent of the infusion used in the present study, has radical-scavenging, anti-inflammatory, neuroprotective and anti-tumorigenic activities [24,25]. Previous studies reported the potential of RA to reduce lipid peroxidation and increase antioxidant enzymes, such as superoxide dismutase and catalase, in models of oxidative stress [26]. Furthermore, it has been demonstrated that RA can reduce levels of 8-oxoguanine, a key marker of oxidative DNA damage and a factor in initiating and promoting carcinogenesis [27]. In addition, the treatment of glioblastoma multiform with RA at a dose of 20 mg/kg has been observed to reduce tumor growth by interfering with intracellular signaling pathways, leading to increased oxidative stress (decreased SOD and CAT levels in tumor tissue), reduced cell proliferation (increased p53 and p21), and induction of apoptosis [28].

The real-time cell viability experiments revealed that the *S. officinalis* aqueous infusion has low cytotoxicity toward rat stomach SM cells. The RTCA software (2.0.0.1301) calculated an average IC_50_ value of 0.34 ± 0.12 mg/mL. The scarce data on the topic of the in vitro cytotoxicity of crude extracts of *Salvia* have shown that the aqueous extract of *S. clandestina*, the methanol extract of *S. miltiorrhiza*, the hydro-ethanol extract of *S. chorassanica* roots, the petroleum ether sub-fractions of the methanol extracts of *S. atropatana* roots, and the methanol extract of *S. rosmarinus* leaves reduced the viability of various cancer cell lines (e.g., MG-63, Glc-82, A549, H460, AGS, MKN-45, MCF-7, PC-3, MDA-MB-231, 184-B5/HER), with IC_50_ values ranging from 4.6 μg/mL to 271.7 μg/mL [29,30].

In another study, 13-epimanoyl oxide isolated from the n-hexane sub-fraction of methanol extracts of *S. macrosiphon* exhibited cytotoxicity against A549, MCF-7, and MDA-MB-231 cancer cell lines (IC_50_ = 11.40, 9.29, and 13.09 μg/mL, respectively), while displaying minimal toxicity toward normal human dermal fibroblasts, with an IC_50_ value of 337.58 μg/mL [31]. Multiple studies have demonstrated that, in cancer cells, phytochemicals from *Salvia* induce plasma membrane and mitochondrial outer membrane permeabilization, leading to cell death via necrosis. Additionally, these phytochemicals increase ROS production, which activates p53, resulting in cell cycle arrest and apoptotic cell death [30]. The observed cytotoxic activities against cancer cells, alongside minor toxicity toward normal cells, make *Salvia* extracts interesting candidates for anticancer therapy. However, the molecular mechanisms of cell death in normal cells caused by these extracts require further investigation.

Our results confirm that the tested *S. officinalis* infusion and some of its components exert an inhibitory effect on the proliferation of SM cells. Experiments on vascular SM cells have demonstrated that salvianolic acid B, one of the active components of *S. miltiorrhiza*, suppresses proliferation by inducing cell cycle arrest and activating the Nrf2/HO-1 pathway [32]. Moreover, danshensu (3-(3,4-dihydroxyphenyl) lactic acid) and salvianolic acid B exhibit antioxidant capabilities [33]. Muscella et al. indicated that exposure of osteosarcoma cells to 60 μg/mL of *S. clandestina* aqueous extract causes cell cycle arrest and p53-dependent apoptosis within 24 h, and they attribute the effect to the synergistic actions of the extract’s components [34]. Our experiments showed cell proliferation inhibition lasting more than 40 h; however, cell monolayer impedance did not decrease, indicating the cell population was not reduced. It is possible that rat stomach SM cells are simply arrested in the cell cycle rather than undergoing apoptosis in response to p53 activation.

The role of ACh and 5-HT in stimulating the proliferation of various cell types is well established. As local signaling molecules, they regulate fundamental functions; cholinergic and serotonergic responses are involved in both cell proliferation and apoptosis. The activation of nicotinic and muscarinic receptors has been shown to exert proliferative and anti-apoptotic effects in many cell types [35]. Conversely, pre-treatment with muscarinic receptor blockers such as 4-DAMP or atropine inhibits proliferative processes [36].

Gastric SM cells are rich in cholinergic and 5-HT receptors that regulate contractility. There are five types of muscarinic receptors: M_1_, M_2_, M_3_, M_4_, and M_5_ [37,38]. Postsynaptic M_3_ receptors are particularly important for regulating contractions, often acting synergistically with M_2_ receptors [39]. Approximately 90% of the body’s 5-HT is found in the gastrointestinal tract [40]. The effects of 5-HT on gastrointestinal SM cells are mediated via 14 receptor subtypes [41], grouped into seven families (5-HT_1_ to 5-HT_7_) [42]. Interaction with these receptors generally induces a contractile effect, primarily mediated by 5-HT_2_ receptors [41].

The effect of the *S. officinalis* infusion on cell viability, combined with the known roles of ACh and 5-HT, raises the question of how this infusion influences these responses in the body. Our studies showed that at concentrations of 0.245, 0.49, and 1.96 mg/mL, the infusion causes a concentration-dependent increase in muscle tone without affecting the frequency of spontaneous phasic contractions. Our analysis of its interactions with ACh and blockers (4-DAMP and atropine) suggests no significant relationship; thus, the *S. officinalis*-induced responses are not cholinergic.

The effects of the *S. officinalis* infusion, ACh, and the cholinergic receptor blockers (4-DAMP and atropine) on the SM samples, and the analysis of their mutual interactions, allow us to state that there is no significant relationship between the effects of the infusion and the cholinergic agents—the infusion-induced responses are not cholinergic in nature.

In contrast, the analysis shows a significant interaction between the effects of the *S. officinalis* infusion and 5-HT. In the presence of the infusion, the effects of 5-HT are significantly reduced. This is clear evidence that the infusion influences 5-HT receptors. A similar conclusion is supported by the significant reduction in the infusion’s effect after receptor blockade with Methysergide.

This strongly suggests that one or more components of the infusion are capable of activating 5-HT receptors, thereby inducing SM contraction in the current experiments. We consider this the primary cause of the infusion-induced contraction. However, the persistence of a reaction in the presence of Methysergide indicates that other components in the infusion exert non-serotonergic effects on SM. This supports the conclusion that the *S. officinalis* infusion induces a complex SM reaction, reflecting the diverse nature of its active compounds.

Beyond its role as a neurotransmitter, 5-HT is involved in numerous body functions and processes: mood, sleep, memory, digestion, nausea, wound healing, bone health, blood clotting, sexual desire, and more. The observed interaction between the components of the *S. officinalis* infusion and the 5-HT receptors, along with its low cytotoxicity and its antioxidant, anti-inflammatory, and cell viability-enhancing properties, suggests its therapeutic potential and makes it a promising subject for further research.

## 4. Materials and Methods

### 4.1. Plants and Extraction

Sage leaves (*S. officinalis* L.) were purchased from a local licensed herbal pharmacy (harvested spring 2024). The botanical identity of the plant material was confirmed in accordance with the European Pharmacopoeia monograph for sage leaves [43]. The dry leaves (50 g) were ground into a fine powder, and 5 g of the powder was infused in 50 mL of boiling deionized water for 30 min with continuous stirring. After filtration of the resulting infusion using Whatmann №1 filter paper, the sample was stored at −20 °C prior to the experiments. The dry matter content of the infusion was determined gravimetrically as the average value of three separate samples.

### 4.2. Chemical Composition of Salvia officinalis Infusion

#### 4.2.1. Total Phenolic Content

The total phenolic content was determined according to the classic Folin and Ciocalteu colorimetric method [44] and expressed as milligrams of gallic acid equivalents (GAEs) per gram dry matter (DM).

#### 4.2.2. Chromatographic Analysis

HPLC-PDA analysis for the identification and quantification of fifteen phenolic compounds was carried out according to a previously developed and validated method [45]. The analysis was carried out using an LC40-PDA (Shimadzu, Kyoto, Japan) equipped with a Shim pack C18 column (3 μm, 150 mm × 4.6 mm) (Shimadzu, Kyoto, Japan). HPLC-grade methanol, acetonitrile, and formic acid were provided by Merck KGaA (Darmstadt, Germany). The chromatographic conditions included a mobile phase, which consisted of 1% formic acid in water (Solvent A), methanol (Solvent B), and acetonitrile (Solvent C) in a gradient elution (as presented in Table 10), a flow rate of 0.5 mL/min, a column temperature of 40 °C, and an injection volume of 10 µL. For the quantification of the compounds, 100 µL of the infusion was diluted up to 5 mL with the mobile phase and filtered through a 0.45 µm syringe filter (Isolab, Eschau, Germany) before injection. The data was analyzed using the LabSolutions software (version 5.118) (Shimadzu, Kyoto, Japan). The measurements were performed in triplicate, with a standard deviation not exceeding 2%.

### 4.3. Evaluation of Antioxidant Capabilities

The antioxidant properties of the infusion were evaluated through three in vitro assays. Free radical scavenging properties were evaluated using the 2,2-diphenyl-1-picrylhydrazyl free radical (DPPH) discoloration method [46] and expressed as μg dry matter of the infusion that scavenges half of the available DPPH. Copper reducing capacity (CUPRAC) was measured according to the original method by Apak et al. and the Trolox equivalency was used as a standard for the antioxidant capacity of such a mixture [47]. Total reducing capacity (TRC) of the infusions was evaluated by the method proposed by Berker [48] and the results were also expressed in Trolox equivalents.

### 4.4. Animals

SM preparations were obtained from the gastric corpus of male Wistar rats (body weight 250–280 g). The animals were sourced from the Animal House of the Medical University in Plovdiv, Bulgaria and maintained under standard laboratory conditions (23–25 °C, 50–55% humidity, 12/12 h light/dark cycle). While the rats had *ad libitum* access to food and water, they were fasted for 24 h prior sample collection.

All experimental procedures were performed in compliance with European Union (Directive 2010/63/EU) and Bulgarian (Directive No. 20/01.11.2012) legislation regarding the use of laboratory animals. This study was conducted under License No. 436/15.07.2025, issued by the Animal Health and Welfare Directorate of the Bulgarian Food Safety Agency (BFSA), https://public-iisr.bfsa.bg/BABHRegsExt/pagesPublic/registers/register11.xhtml?locale=en (accessed on 11 June 2026)).

### 4.5. Smooth Muscle Cell Culture, Cell Viability Assay and Treatment Procedures

The SM layer from the gastric corpus of rats (n = 2 animals) was obtained in situ with the mucosa removed. Primary SM cells were isolated as previously described [38]. Briefly, the cells were morphologically characterized as elongated, spindle-shaped, bipolar, and containing a centrally located nucleus. They exhibited the characteristic “hills and valleys” growth pattern of SM cells. Cells from passages 3 to 5 were used for the experiments. Cells were cultured in growth medium (DMEM) supplemented with 20% fetal bovine serum, 100 U/mL penicillin, 100 μg/mL streptomycin, and 5 μg/mL amphotericin B, in a humidified incubator at 37 °C and 5% CO_2_.

Cell viabilities were observed in real time using the xCELLigence RTCA-DP system (ACEA Biosciences, San Diego, CA, USA). For this purpose, cells were seeded on a 16-well plate (E-Plate VIEW 16, ACEA Biosciences, cat # 300600880) at a density of 2 × 10^4^ cells/cm^2^ and placed in the xCELLigence system. Impedance was measured continuously and non-invasively, enabling the monitoring of changes in cell number, cell–substrate attachment quality, proliferation rate, and cell monolayer development. The condition of the cell monolayer during the experiment was also observed under an inverted light microscope.

Cells grown to 80–90% confluence were treated with different concentrations of *S. officinalis* infusion. For this purpose, plates were removed from the xCELLigence system, and the growth medium in the wells was replaced with growth medium containing the *S. officinalis* infusion. The xCELLigence RTCA-DP system converted the impedance measurements (changes in electrical resistance) of adherent cells into a CI. Measurements were taken every 30 min over a period of 2–3 days. The cell growth curve was plotted as the dependence of the CI on the culture time. The resulting curves were used to determine IC_50_ values. Furthermore, using the xCELLigence RTCA-DP software (version 2.0.0.1301), we determined the average DT for each concentration. The obtained results could be either positive (the amount of cells in the monolayer is increasing) or negative (the amount of cells in the monolayer is decreasing).

### 4.6. Isometric Registration of Smooth Muscle Contractile Reactivity

Ten Wistar rats were used for the in vitro studies to elucidate the changes in the mechanical activity of stomach SM upon treatment with the studied plant infusions and other biologically active substances. The animals were decapitated, and, immediately after that, stomach SM preparations (corpus) were excised in situ, separating only the muscle tissue without the mucosa. Twenty-four hours before in vitro experiments, the animals were deprived of food. At the beginning of the experiments, they were euthanized by an overdose of anesthesia (xylazine 10 mg/kg and ketamine 100 mg/kg). SM preparations were obtained from the circular muscle layer of the gastric corpus, 1.0 mm wide and 10 mm long.

The preparations were firmly attached to a glass holder at one end and connected via surgical thread at the other to a force transducer, part of Tissue Organ Bath System (159920, Radnoti, AD Instruments Limited, Oxford, UK). The electrical signal obtained from them was amplified using an amplifier Octal Bridge Amp (FE228, Radnoti, AD Instruments Limited, UK).

The registration of mechanical activity was performed using an isometric method as previously described [38]. Briefly, the initial mechanical loading for the preparations achieved by stretching corresponded to a tension force of 7 mN, with an adaptation period of 60 min. Changes in spontaneous mechanical activity and tone caused by exposure to different biologically active substances were recorded with PowerLab data acqusition system 8/35 and LabChart analysis software, version 8.1.16 (both developed by AD Instruments Limited, UK).

### 4.7. Statistical Methods

Statistical analysis was performed using SPSS software, version 17.0 (SPSS Inc., Chicago, IL, USA). The analysis began with determination of data distribution using the Shapiro–Wilk normality test. The data obtained are expressed as mean ± standard error of mean. The number of test points (sample sizes) used in each experiment is indicated by n. Statistical differences were tested using paired *t*-test for the isolated tissue samples due to the fact that the same samples were used as control (pre-treatment) and treatment. A U-test (Mann–Whitney) was used for the cell cultures. Probability of *p* < 0.05 was considered significant.

## 5. Conclusions

In summary, the aqueous infusion of *S. officinalis* demonstrates a clear concentration-dependent effect on gastric SM cells following 48–72 h of exposure. At higher concentrations (2.45–0.25 mg/mL), the infusion exhibits cytotoxic properties and inhibits cell proliferation, while lower concentrations (below 0.25 mg/mL) show minimal or no observable effect on cell viability. The functional assays on the isolated rat gastric SM cells further revealed a dose-dependent enhancement of both tonic and phasic contractile activity. Interestingly, these contractile responses were maintained in the presence of muscarinic antagonists, suggesting a mechanism of action independent of this classical pathway. There is a decreased tonic response to *S. officinalis* after the application of a serotonergic receptor antagonist, suggesting the partial involvement of serotonergic pathway.

These results support the traditional medicinal use of *S. officinalis* and underscore its potential as a modulator of SM function and as a source of bioactive antioxidant compounds. Further research is necessary to elucidate the exact molecular mechanisms involved and to explore its possible therapeutic applications in gastrointestinal and neurodegenerative conditions.

## Figures and Tables

**Table 1:** Phenolic compounds identified and quantified in *Salvia officinalis* infusion. The results are presented as mg phenolic compound per g dry matter.

Compound	Concentration, mg/g
Chlorogenic acid	NI
Catechin	NI
Caffeic acid	3.00
Rutin	3.58
p-Coumaric acid	NI
Ferulic acid	NI
Hesperidin	1.84
Rosmarinic acid	38.16
Salicylic acid	NI
Quercetin	<LQ
Luteolin	<LQ
Kaempferol	NI
Apigenin	NI
Casticin	NI
Acacetin	<LQ

NI—not identified; <LQ—below the limit of quantification.

**Table 2:** In vitro antioxidant capacity of infusion obtained from *S. officinalis.* The results for the DPPH assay are presented as dry mass of infusion capable of scavenging 50% of the available DPPH radicals; the results for other two methods are presented as Trolox equivalents (TEs).

Antioxidant Properties	*S. officinalis*
Radical-scavenging activity (DPPH, IC_50_, µg/mL)	21.77 ± 0.98
Total reducing capacity (TE mmol/g)	1.92 ± 0.01
Cupric ion reducing antioxidant capacity assay (CUPRAC, TE mmol/g)	2.32 ± 0.04

**Table 3:** Maximum value of tonic contractions caused by successive exposures to different concentrations of *S. officinalis* infusion; n = 4 for each exposure. Results are presented as mean ± SEM.

Concentration of *S. officinalis*	0.245 mg/mL	0.49 mg/mL	1.96 mg/mL
Mean value of tonic contraction [mN]	2.28 ± 0.30	3.64 ± 0.41	5.20 ± 0.52

**Table 4:** Amplitude (strength) of spontaneous phasic contractions of SM samples (n = 4) after treatment with 1.96 mg/mL of the infusion. Results are presented as mean ± SEM of 5 consecutive phasic contractions after 3 and 30 min.

Contraction Before Application, [mN]	After *S. officinalis* Infusion, [mN]	
	3 min After Application	30 min After Application
2.96 ± 0.28	3.47 ± 0.25	0.66 ± 0.10 *

A comparison with the baseline value (auto-control) before exposure to *S. officinalis* was done; *—*p* < 0.05.

**Table 5:** Strength of ACh-induced tonic contractions for the control and in the presence of *S. officinalis* infusion (n = 9).

Substance	ACh 1 × 10^−6^ mol/L	*S. officinalis* 1.96 mg/mL + ACh 1 × 10^−6^ mol/L
Contraction, [mN]	9.10 ± 0.79	9.20 ± 0.39

**Table 6:** *S. officinalis* (1.96 mg/mL)-induced contractions of SM samples pretreated with 1.10^−6^ mol/L ACh (n = 7).

Substance	ACh 1 × 10^−6^ mol/L	ACh 1 × 10^−6^ mol/L + *S. officinalis*
Contraction, [mN]	8.54 ± 0.34	9.48 ± 0.31 *

A comparison with the baseline value (auto-control of ACh-induced contraction) before exposure to *S. officinalis* was done; *—*p* < 0.05.

**Table 7:** *S. officinalis* (1.96 mg/mL)-induced SM contractions under the condition of blocked m-cholinergic receptors (n = 7).

Substance	*S. officinalis*	4-DAMP 1 × 10^−6^ mol/L + *S. officinalis*	Atropine 1 × 10^−6^ mol/L + *S. officinalis*
Contraction, [mN]	3.74 ± 0.39	2.97 ± 0.41	3.17 ± 0.38

**Table 8:** Effect of 1.96 mg/mL *S. officinalis* on the strength (amplitude) of 5-HT-induced contractions (n = 8).

Substance	5-HT 1 × 10^−6^ mol/L	*S. officinalis* + 5-HT 1 × 10^−6^ mol/L
Contraction, [mN]	8.58 ± 0.79	4.11 ± 0.39 *

A comparison with the baseline value (auto-control of 5-HT-induced contraction) before exposure to *S. officinalis* was done; *—*p* < 0.05.

**Table 9:** Potency of 1.96 mg/mL infusion of *S. officinalis*-induced contractions in the background of Methysergide 3 × 10^−7^ mol/L (n = 4).

Substance	*S. officinalis*	Methysergide 3 × 10^−7^ mol/L + *S. officinalis*
Contraction, [mN]	4.45 ± 0.16	1.51 ± 0.20 *

A comparison with the baseline value (auto-control of *S. officinalis*) after exposure to Methysergide 3 × 10^−7^ mol/L was done; *—*p* < 0.05.

**Table 10:** HPLC mobile phase gradient used for the analysis of flavonoids and phenolic acids.

Time (min)	Solvent A (%)	Solvent B (%)	Solvent C (%)
0	75	5	20
15	60	40	0
24	50	50	0
40	50	50	0
60	20	80	0
70	75	5	20

## Data Availability

The original contributions presented in this study are included in the article. Further inquiries can be directed to the corresponding author.

## Abbreviations

The following abbreviations are used in this manuscript:

ACh	Acetylcholine
CI	Cell Index
CUPRAC	Copper reducing capacity
DT	Doubling time
DPPH	2,2-diphenyl-1-picrylhydrazyl free radical assay
HPLC-PDA	High performance liquid chromatography with photodiode array detector
RA	Rosmarinic acid
RTCA	Real-time cell analyzer
ROS	Reactive oxygen species
TRC	Total reducing capacity
SM	Smooth muscle/s
4-DAMP	1,1-dimethyl-4-diphenylacetoxypiperidinium iodide
5-HT	5-hydroxytryptamine
